# Bayou virus-associated hantavirus pulmonary syndrome in Eastern Texas: identification of the rice rat, Oryzomys palustris, as reservoir host.

**DOI:** 10.3201/eid0401.980115

**Published:** 1998

**Authors:** N Torrez-Martinez, M Bharadwaj, D Goade, J Delury, P Moran, B Hicks, B Nix, J L Davis, B Hjelle

**Affiliations:** University of New Mexico School of Medicine, Albuquerque, USA.

## Abstract

We describe the third known case of hantavirus pulmonary syndrome (HPS) due to Bayou virus, from Jefferson County, Texas. By using molecular epidemiologic methods, we show that rice rats (Oryzomys palustris) are frequently infected with Bayou virus and that viral RNA sequences from HPS patients are similar to those from nearby rice rats. Bayou virus is associated with O. palustris; this rodent appears to be its predominant reservoir host.


### Bayou Virus-Associated Hantavirus Pulmonary Syndrome in Eastern Texas: Identification of the Rice Rat, *Oryzomys palustris*, as Reservoir Host

We describe the third known case of hantavirus pulmonary syndrome (HPS) due to Bayou virus, from Jefferson County, Texas. By using molecular epidemiologic methods, we show that rice rats (*Oryzomys palustris*) are frequently infected with Bayou virus and that viral RNA sequences from HPS patients are similar to those from nearby rice rats. Bayou virus is associated with *O. palustris;* this rodent appears to be its predominant reservoir host.

The 1993 discovery of a clinically distinct form of hantavirus disease now known as hantavirus pulmonary syndrome (HPS) highlighted the existence of a previously unrecognized complex of New World hantaviruses, each of which is associated with a specific rodent of the subfamily Sigmodontinae, family Muridae. The prototype of this complex is the Sin Nombre (SN) virus of the deer mouse, *Peromyscus maniculatus*. SN virus, which occurs most frequently in the western United States and Canada, is responsible for more than 95% of cases of HPS in North America. The case-fatality rate of HPS is nearly 50% (1).

Three viruses other than SN have been associated with HPS in North America. All cases associated with viruses other than SN have occurred outside the range of the deer mouse. Two cases have been linked to New York virus in the northeastern United States; the white-footed mouse, *P. leucopus*, was the only hantavirus carrier in the area where the infections were contracted. The cotton rat (*Sigmodon hispidus*)–associated Black Creek Canal virus is believed to have caused a single case in southern Florida, albeit without molecular confirmation. Bayou (BAY) virus has been identified from cDNA sequences amplified from the necropsied tissues or the blood of patients from Louisiana and eastern Texas (1-6).

Evidence implicates the rice rat, *Oryzomys palustris*, as the carrier of BAY virus. In a survey of archived rodent tissue samples obtained from species indigenous to Louisiana, only *O. palustris* samples were positive for hantavirus antibodies, and cDNAs of a BAY-like virus were amplified from two of those samples (7). A few additional BAY virus–RNA-positive rice rat samples have appeared in subsequent studies (6). However, no substantial rodent collections have been conducted in association with BAY virus HPS cases, and no direct molecular association has been found between human BAY virus genomic sequences and those obtained from samples from rodents trapped at possible sites of human infection. The purpose of this study was to identify the carrier rodents associated with two human cases of BAY virus infections and to further characterize the clinical consequences of human infection with BAY virus.

### Diagnostic Studies

Initial serologic investigations to detect antibodies to hantavirus used a sandwich μ-capture enzyme-linked immunosorbent assay (ELISA) as well as an immunoglobulin (Ig) G ELISA, with recombinant SN virus nucleocapsid (N) protein as the target antigen (8). Confirmatory testing with a larger array of recombinant-expressed viral N antigens was conducted by using strip immunoblot and Western blot formats (9-11). The strip immunoblot assay incorporates five membrane-bound antigens (recombinant-expressed SN virus N and G1, recombinant-expressed Seoul virus N, and synthetic peptides of SN N and G1)(11). Antibodies reactive to these antigens are detected with an anti-human immunoglobulin heavy+light chain conjugate, and the assay thus has some sensitivity for IgM but greater sensitivity for IgG. The Western blot uses both anti-IgG and anti-IgM conjugates in separate assays (9).

Western blot studies used a panel of affinity-purified, full-length N antigens produced in *Escherichia coli* in fusion with the phage T7 gene 10 protein in the pET23b vector (10). Recombinant N antigens were purified over metal chelation affinity columns through a polyhistidine tag on the C-terminus of each protein. The recombinant proteins were produced in an isogenic background and loaded at 500ng/lane before sodium dodecyl sulfate-polyacrylamide gel electrophoresis separation and electrophoretic transfer. The antigens were derived from the following hantaviruses: SN (3H226); BAY (OP-LA-475); Rio Mamoré (OM-556), Muleshoe (SH-Tx-339), Puumala (P360), and Seoul (80/39).

### Rodent Collection and Processing

Since the two BAY virus-HPS cases from Texas occurred in neighboring cities, this study includes rodent samples collected in the investigation of both case P/Tx (6) and the case described here, T/Tx. Sherman live-traps were used to collect rodents in Jefferson County and neighboring Orange County. Heart blood samples were screened by a recombinant SN virus N antigen ELISA (8).

### Viral Sequencing and Phylogenetic Analyses

Human peripheral blood mononuclear cells (2 x $10^6$) and rodent lung, kidney, and spleen samples (~200 mg total) were used to make RNA (6,10). A standard set of partially nested primers in the viral S segment was used in reverse transcription-polymerase chain reaction (RT-PCR) analyses of the RNA (6,10). This procedure produces a 442-nucleotide (nt) amplification product; 397 nt of that sequence is internal to the primers. The PCR products were subjected to direct DNA sequencing with an ABI 377 automatic sequencer. Phylogenetic trees were constructed from the 397 nt of informative sequence by using maximum parsimony with PAUP 3.1 software (12).

### A Third Case of HPS Due to BAY Virus

Patient T/Tx is a 54-year-old African-American man from Jefferson County, Texas. A heavy tobacco user, he has a more than 100 pack -years* smoking history and chronic shortness of breath. Approximately 2 weeks before admission, his spouse noted that he had increased fatigue and somnolence. By August 20, 1996, his symptoms worsened, and he sought medical care. He visited a local emergency room with complaints of increasing shortness of breath and low-grade fever for 2 days. Hospital personnel believed that he had probable chronic obstructive pulmonary disease and chronic bronchitis. Results of a physical examination of the patient at that time were unremarkable, and a chest radiograph was interpreted as hyperinflated but without infiltrates (Figure 1A). He was afebrile (37°C) in the emergency room. Laboratory analysis showed a white blood cell count of 4,080/µl and a platelet count of 122,000/µl. He was given amoxicillin and bronchodilators and was discharged.

On August 21, the patient returned to the emergency room with persistent and worsening dyspnea. He also had flulike symptoms, including myalgias, and had had a temperature of 39.4°C. Physical examination revealed bilateral rhonchi. He had a blood pressure of 90/60, pulse of 96/min, respiratory rate of 16/min, and an oral temperature of 37°C. Laboratory analysis noted an increase in his white blood cell count to 7,700/µl with a left shift and a decrease in platelet count to 65,000/µl. The patient was admitted to the hospital with a preliminary diagnosis of chronic obstructive pulmonary disease exacerbation and possible pneumonia. He was given broad-spectrum antibiotics, methylprednisolone, aminophylline, and ipatronium nebulizer treatments.

The patient's condition deteriorated rapidly over the next 24 hours, and he was severely short of breath by the second hospital day. He was also febrile and was transferred to the intensive care unit to manage his worsening shortness of breath. His oxygen demand increased, and on the third hospital day, the patient required endotracheal intubation. A physical examination found bilateral expiratory wheezing and bibasilar rales. A chest radiograph showed increasing bilateral interstitial and alveolar infiltrates. By the fourth hospital day, the patient required 100% oxygen and positive end-expiratory pressure support. A chest radiograph again showed interstitial and alveolar infiltrates with peribronchial cuffing and pleural effusion. His fluid intake was restricted to manage his pulmonary edema; he briefly required dopamine for hypotension. Blood and urine cultures remained negative. Sputum Gram stain showed a moderate number

*Pack-year = no. packs/day x no. years

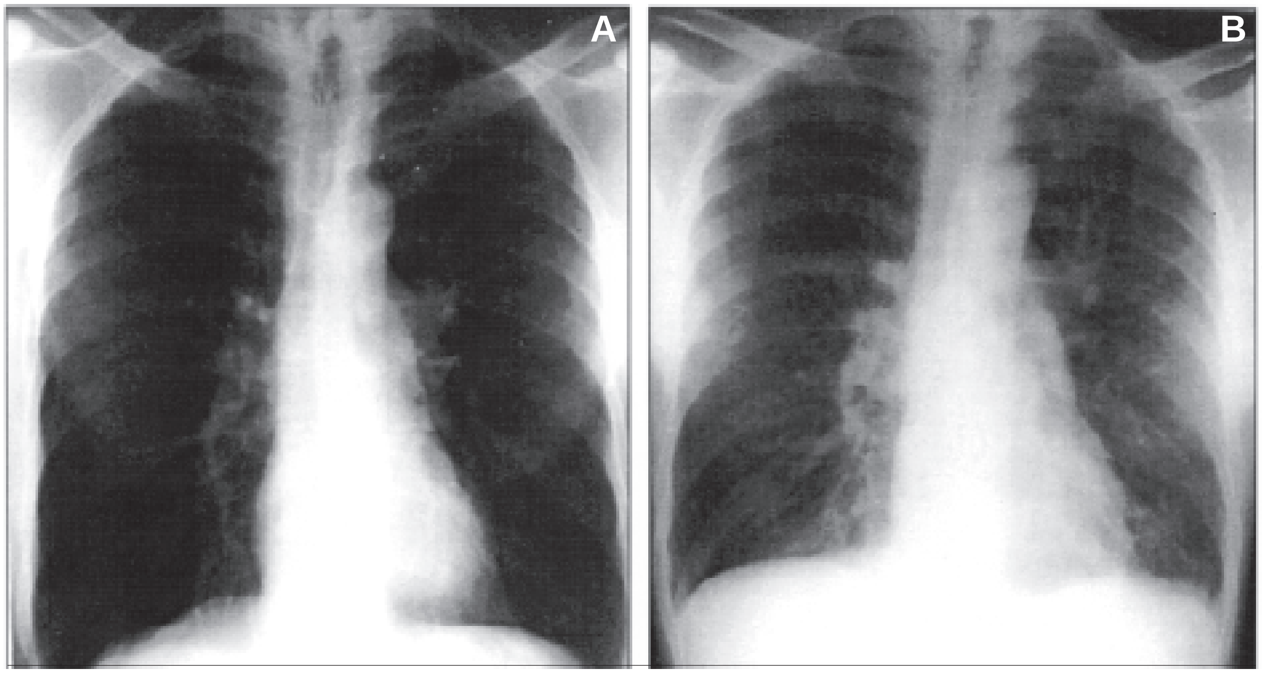

Figure 1. Chest radiographs of patient T/Tx at hospital admission on August 20, 1996 (Panel A), and during a period of increasing respiratory distress on August 22, 1996 (Panel B). Note the diffuse interstitial infiltrate and peribronchial cuffing that developed over that interval.

of white blood cells and few budding yeast; culture showed no pathogens. Serologic tests for mycoplasma and *Legionella* were negative.

By the fifth hospital day, the patient's condition had begun to improve. Over the next 5 days the fever diminished, the pulmonary edema improved, and requirement for supplemental oxygen decreased. The endotracheal tube was removed on the 10th day of hospitalization; by the 14th day, the patient was discharged—ambulatory, afebrile, and no longer requiring supplemental oxygen.

The hematologic laboratory findings were generally most abnormal when the patient was most acutely ill clinically; the peak serum chemistry abnormalities trailed the clinical illness by several days. The maximum white blood cell count was 31,700/µl on August 23, with a differential count of 7% lymphocytes (including the characteristic plasmacytoid forms; [13]), 44% mature neutrophils, 35% band neutrophils, 3% metamyelocytes, and 2% myelocytes. The platelet count reached a nadir of 19,000/µl on that day, and the hemoglobin reached a maximum of 17.2 g/dL on August 22. The serum creatinine was 0.6 mg/dL on August 24 but peaked at 1.9 mg/dL on August 25. This mild azotemia was associated with proteinuria (300 mg/dL in a spot sample obtained on August 22). The serum enzymes creatine kinase and lactic dehydrogenase peaked on August 27 at 917 units/L and 933 units/L, respectively. Fractionation of the creatine kinase and lactic dehydrogenase isoenzyme forms were not supportive of myocardial injury as a cause for their elevation. Serum levels of the liver transaminases were modestly elevated.

### Environmental Assessment and Rodent Collection

The patient worked as a laborer at a railroad construction company. For 2 months before his illness, he replaced old rail lines at three industrial plants (Beaumont, Neches River, and Orange) in Orange and Jefferson Counties and cleaned the yard and garage at work headquarters. He manually removed and replaced tracks and ties serving warehouses, transport pipes, and scrap metal piles.

The Beaumont and Orange work sites were chemical plants. All rail work occurred within the confines of the mowed plant complex. Some of the track sites were next to drainage ditches and canals; others coursed by warehouses reported by plant employees to have rats. The patient had not seen any rodents in the months before becoming ill, but he remembered rodent droppings at a wooden crew trailer at the Beaumont plant. All rodents trapped at these plants tested negative for hantavirus antibodies.

The Neches River work site was a recycling plant situated for commerce by rail and ship. The rails were within several meters of permanent bayous and swamps, piles of scrap metal, and the Neches River. The vegetation near the water was dense with trees and undergrowth. Truck and train traffic at the plant created a substantial dust problem. The site contained stray dogs and cats as well as a variety of wildlife, including snakes and alligators. Although trapped heavily, few rodents were collected from near the tracks and adjacent swamps. All rodents trapped at this facility were negative for hantavirus antibodies.

Although the patient lived in an older pier-and-beam home, he and his wife saw no evidence of rodent infestation. A Texas Department of Health investigator verified the absence of rodents and rodent excreta. Although traps were set, no rodents were collected at the residence.

During the 2 months before he became ill, the patient visited a casino boat in Louisiana and fished three times from the Pleasure Island jetty in southern Jefferson County. The jetty had large rock boulders at the water's edge and an asphalt road along its length. Sand and dense 6-foot high grasses covered the remainder of the jetty. Household trash and fishing debris were scattered throughout the area. Most rodents collected by the Texas Department of Health investigation team during this investigation were from this jetty; and most were *O. palustris*, including three seropositive specimens (Area 1, Figure 2).

Other sites were chosen between the patient's work sites and home because their habitats were likely to support rodents. Rice rat habitats (permanent water and grasses) were among those targeted, because the first Jefferson County hantavirus investigation documented the Bayou strain of hantavirus in *O. palustris* (6). The Table and Figure 2 show the location and species of each rodent collected and the number of seropositive specimens in each of four targeted areas. Four hantavirus antibody-positive rodents were trapped at these miscellaneous locations during the investigation of the current HPS case. Two of the seropositive samples were from Area 1 of Figure 2 (not the jetty), and two were in Area 2. Area 2 yielded two seropositive rodents during the first Texas BAY virus case investigation (6).

### Diagnostic Studies

The IgG ELISA assay for SN virus antibodies in patient T/Tx's serum was negative, but the IgM test was positive at a 1:6400 dilution (8). The strip immunoblot assay showed antibody reactivities typically seen with HPS caused by a hantavirus other than SN virus, with 2+ reactivity to the immunodominant SN virus N peptide and 4+ reactivity to the full-length recombinant N protein (11). There was no reactivity to the SN virus G1 antigen, either in peptide or recombinant form, or to Seoul virus recombinant N protein (data not shown). Antibody reactivity to the SN virus G1 antigen is specific for infection with SN virus.

Western blot analysis of patient T/Tx's serum showed IgG antibodies to hantavirus N proteins

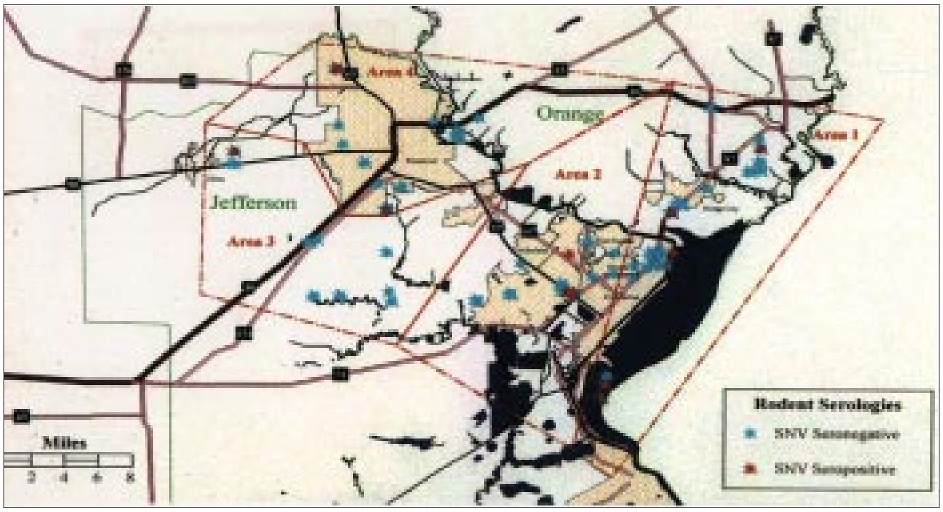

Figure 2. Location of rodents trapped during investigations related to patient P/Tx (March 1996; [6]) and patient T/Tx (October 1996; this report). The locations in which rodents were collected are broadly classified into four areas, designated 1 through 4.

Table. Rodents collected in association with two cases of Bayou virus-hantavirus pulmonary syndrome in Jefferson and Orange Counties, Texas, March and October 1996

| | No. specimens collected (no. seropositive) | | | | | No. PCR[b] tested |
|---|---|---|---|---|---|---|
| Species | Area 1[a] | Area 2 | Area 3 | Area 4 | Tot. | (no. pos.) |
| *Baiomys* | 0 | 0 | 1 | 3 | 4 | 0 |
| *taylori* | | | (0) | (0) | (0) | |
| *Mus* | 30 | 21 | 12 | 26 | 88 | 1 |
| *musculus* | (0) | (0) | (1) | (0) | (1) | (0) |
| *Ochrotomys* | 0 | 0 | 0 | 1 | 1 | 0 |
| *nuttali* | | | | (0) | (0) | |
| *Oryzomys* | 45 | 20 | 3 | 8 | 76 | 15 |
| *palustris* | (7) | (5) | (0) | (3) | (15) | (14) |
| *Peromyscus* | 0 | 3 | 0 | 0 | 3 | 0 |
| *leucopus* | | (0) | | | (0) | |
| *Rattus* | 1 | 1 | 0 | 1 | 3 | 0 |
| *norvegicus* | (0) | (0) | | (0) | (0) | |
| *Rattus* | 21 | 8 | 0 | 1 | 30 | 0 |
| *rattus* | (0) | (0) | | (0) | (0) | |
| *Reithrodontomys* | 0 | 7 | 0 | 7 | 14 | 0 |
| *fulvescens* | | (0) | | (0) | (0) | |
| *Reithrodontomys* | 1 | 0 | 0 | 2 | 3 | 0 |
| *humulis* | (0) | | | (0) | (0) | |
| *Sigmodon* | 95 | 5 | 11 | 4 | 114 | 1 |
| *hispidus* | (0) | (0) | (0) | (1) | (1) | (1) |

[a]Refer to Figure 2 for map areas.
[b]PCR=polymerase chain reaction.

(Figure 3, Panel A). Although the most intense staining was observed with the homologous (BAY virus) antigen, varying cross-reactivity was present against the N antigens of Muleshoe, SN, Rio Mamoré, and Puumala viruses but not of Seoul virus. A similar pattern was observed when another blot membrane was probed with an anti-human IgM conjugate, except that reactivities were somewhat more intense (data not shown). Serum samples of seropositive rice rats and deer mice showed similar cross-reactivities, although the intensity of reactivity to a particular N antigen was related to the sequence similarity between the membrane-bound antigen and the virus against which the antibodies were directed (Figure 3, Panels B and C).

RT-PCR analyses and sequencing of the cDNA product of the patient's blood sample identified definitively BAY virus as the etiologic agent. When the 442 nt viral S segment amplification product from patient T/Tx was compared with the homologous sequence of other hantaviruses, it was closely similar to previously described BAY viruses from Louisiana and Texas (Figure 4). Nine viral sequences from seropositive *O. palustris* and one from a seropositive *S. hispidus*

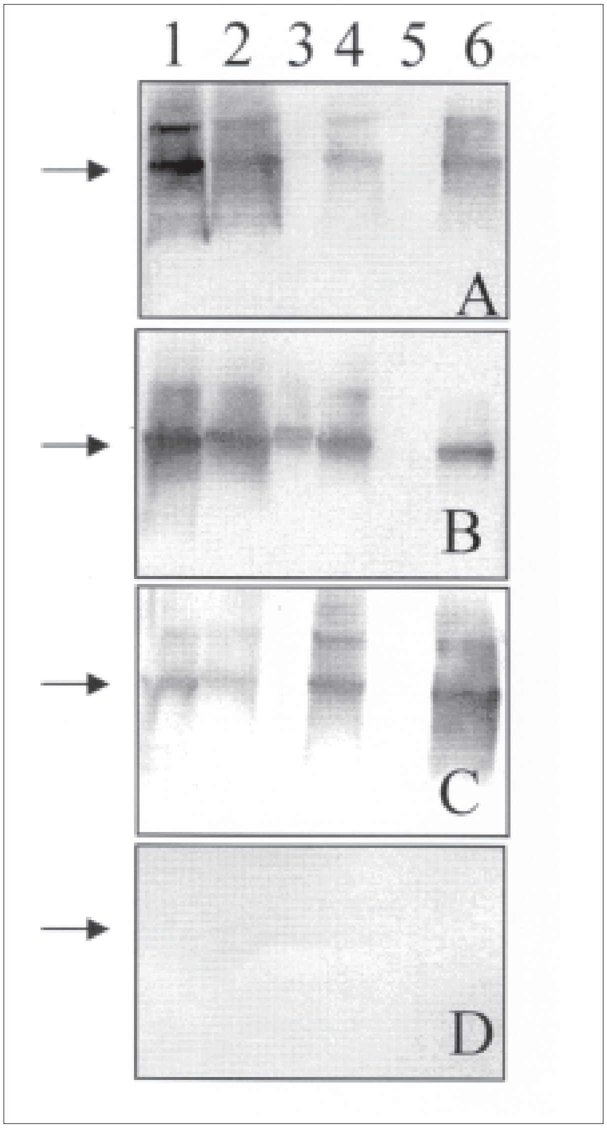

Figure 3. Western blot assay for detecting IgG antibodies in patient and rodent blood samples. A 1:500 dilution of serum was used to probe Western blots containing equimolar amounts of recombinant-expressed N antigens of various hantaviruses: 1) Bayou; 2) Muleshoe; 3) Puumala; 4) Rio Mamoré; 5) Seoul; and 6) Sin Nombre. Serum samples are directed against specific hantaviruses as verified by reverse transcription-PCR and sequence analyses: (A) patient T/Tx, Bayou virus; (B) Bayou virus-seropositive *Oryzomys palustris* specimen #505, collected in Jefferson County, Texas; (C) a Sin Nombre–seropositive deer mouse, collected in California; (D), a negative control (hantavirus-seronegative) deer mouse. Antibodies bound to the solid-phase antigens were detected with alkaline phosphatase-conjugated anti-human IgG (A) or with anti-*Peromyscus leucopus* IgG (B-D). The arrow indicates the migration of the full-length T7-viral N antigen, ~55kDa.

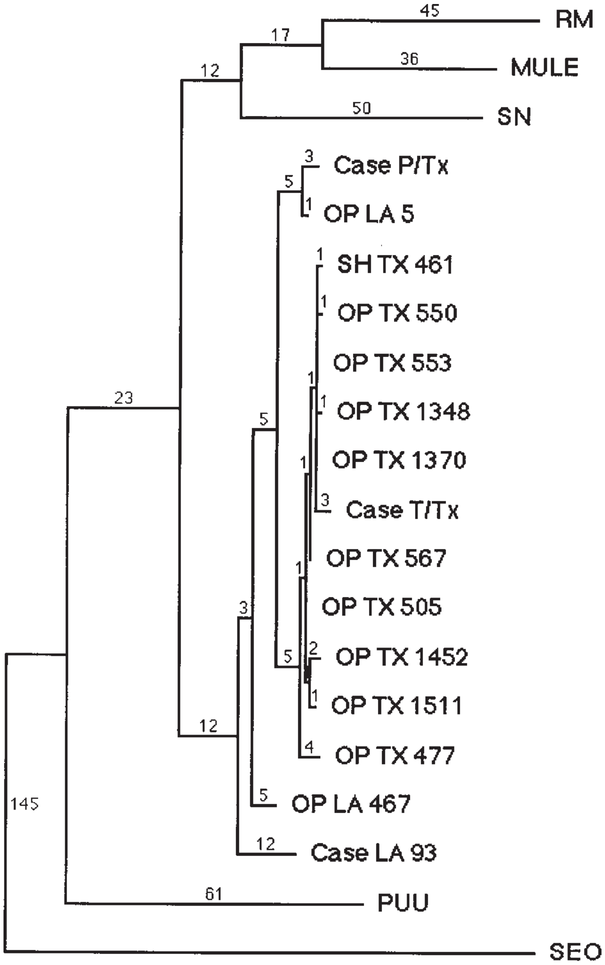

Figure 4. Unweighted maximum parsimony tree produced by PAUP 3.1 software comparing a 397 nt portion of the hantavirus S genomic segments (residues 207 to 603) of patients and rodents infected with Bayou virus. A selection of other prototypical hantavirus sequences, each of which was compared antigenically in Figure 2, was included for comparison. Other hantaviruses are abbreviated as follows: RM=Rio Mamoré; MULE=Muleshoe; SN= Sin Nombre; PUU=Puumala; and SEO=Seoul. Human-derived Bayou virus sequences are indicated by Case, and rodent sequences by OP (*Oryzomys palustris*) or SH (*Sigmodon hispidus*). TX=Texas, LA=Louisiana.

from Jefferson County, Texas, and Cameron and Terrebonne Parishes, Louisiana, were compared with those of patients T/Tx and P/Tx and with that of a 1993 case-patient from northern Louisiana (Case-LA-93, Figure 4).

The sequences of all the Jefferson County rodents and patient T/Tx were closely related to each other, differing by 1 to 10 nt (0.25% to 2.5% of 397 evaluable residues) in pairwise comparisons. Four rodent-derived viral sequences differed from that of the patient T/Tx virus by 4 nt (1%), and all were from rodents collected in Jefferson County.

Except for a brief visit to a Louisiana casino, patient T/Tx reported that he had not traveled outside east Texas in the 2 months before his illness. The generally close genetic similarity among the viral sequences from east Texas supports the hypothesis that patient T/Tx became infected in that region (14). Potential exposure sites, as defined by his travel history, were too numerous to allow us to identify a more precise site of infection. No rodent sequence was completely identical to that of patient T/Tx.

The sequence obtained from patient P/Tx (6), who was thought to have been infected in Jefferson County, was not closely aligned with any eastern Texas rodent sequence or with that of patient T/Tx. The closest of the 11 eastern Texas viral sequences differed from that of patient P/Tx at 13 residues (3.3%). One sequence available from previous studies was far closer to that of patient P/Tx, differing at only four residues (1%). This sequence was obtained from a rice rat (OP-LA-5) collected in Cameron Parish, Louisiana, approximately 30 km from the former residence of patient P/Tx. Although patient P/Tx initially reported no travel back to his former residence (where his mother still lived) in the 6 weeks before his illness (6), he later was not certain about his travel history in the weeks before his illness, stating that he might have visited his mother in Louisiana in the weeks before his illness.

In previous studies, *O. palustris* was tentatively implicated as the predominant rodent reservoir for BAY virus, but the sample sizes were small, and specific cases of HPS were not linked to the occurrence of BAY virus-infected rodents at the presumed sites of infection (6,7). For this study, analysis of a substantial collection of rodents collected in the vicinity of sites of human infection showed that *O. palustris* is the species with the highest hantavirus seroprevalence. Furthermore, the hantavirus genotypes in circulation in the eastern Texas/western Louisiana area were related to those of two human case-patients from the area, and all but one of those rodent-derived sequences came from *O. palustris*.

Rice rats are found in wetlands and marshes from Texas throughout the southeastern United States, extending north as far as New Jersey on the eastern seaboard. Like all carriers of viruses associated with HPS, *O. palustris* is a member of the subfamily Sigmodontinae, family Muridae. Although only a single species exists in the United States, the tribe Oryzomyini has scores of species in Latin America, where HPS is increasingly recognized as an important zoonotic disease. Viruses similar to BAY virus have been recognized recently in members of the Oryzomyini from Bolivia and Argentina, and in some cases these rodent-borne viruses have been linked to HPS in humans (1,15).

The possibility that the genetically distinct clade of viruses associated with oryzomine and *Sigmodon* rodents produces a disease that is qualitatively different from SN virus-associated HPS (16) has been raised repeatedly as new cases have been identified (5,6,17). Although patient T/Tx had a relatively mild course of HPS, his serum creatinine, urine protein concentration, and creatine kinase were nevertheless slightly elevated. Both the elevation of creatine kinase and chemical evidence of myositis have been observed in HPS caused by viruses of the oryzomine and *Sigmodon* clade, but much less commonly in SN virus infection (1). Further studies are needed to determine the basis for the variant clinical symptoms of HPS in these patients.

### Acknowledgments

We thank G. Haigh, McNeese State College, for donating the rice rat specimen OP-LA-5; J. Griffith for technical assistance with sequencing; and W. George, St. Elizabeth Hospital, for first recognizing HPS as a possible cause of patient T/Tx's illness. This study was supported by DHHS grant RO1 AI36336 (to B.H.).

**Norah Torrez-Martinez,* Mausumi Bharadwaj,* Diane Goade,* John Delury,† Peggy Moran,* Bradley Hicks,† Beverlee Nix,‡ James L. Davis,¶ and Brian Hjelle***

*University of New Mexico School of Medicine, Albuquerque, New Mexico, USA; †Texas Department of Health, Austin, Texas, USA; ‡Texas Department of Health, Houston, Texas, USA; and ¶St. Elizabeth's Hospital, Beaumont, Texas, USA.

### References

1. Schmaljohn C, Hjelle B. Hantaviruses: a global disease problem. Emerg Infect Dis 1997;3:95-104.
2. Song J-W, Baek L-J, Gajdusek DC, Yanagihara R, Gavrilovskaya I, Luft BJ, et al. Isolation of pathogenic hantavirus from white footed mouse (*Peromyscus leucopus*). Lancet 1994;344:1637.
3. Rollin PE, Ksiazek TG, Elliott LH, Ravkov EV, Martin ML, Morzunov S, et al. Isolation of Black Creek Canal virus, a new hantavirus from *Sigmodon hispidus* in Florida. J Med Virol 1995;46:35-9.
4. Morzunov SP, Feldmann H, Spiropoulou CF, Semenova VA, Rollin PE, Ksiazek TG, et al. A newly recognized virus associated with a fatal case of hantavirus pulmonary syndrome in Louisiana. J Virol 1995;69:1980-3.
5. Khan AS, Spiropoulou CF, Morzunov S, Zaki SR, Kohn MA, Nawas SR, et al. Fatal illness associated with a new hantavirus in Louisiana. J Med Virol 1995;46:281-6.
6. Hjelle B, Goade D, Torrez-Martinez N, Lang-Williams M, Kim J, Harris RL, Rawlings JA. Hantavirus pulmonary syndrome, renal insufficiency and myositis associated with infection by Bayou hantavirus. Clin Infect Dis 1996;23:495-500.
7. Torrez-Martinez N, Hjelle B. Enzootic of Bayou hantavirus in rice rats (*Oryzomys palustris*) in 1983. Lancet 1995;346:780-1.
8. Ksiazek TG, Peters CJ, Rollin PE, Zaki S, Nichol S, Spiropoulou C, et al. Identification of a new North American hantavirus that causes acute pulmonary insufficiency. Am J Trop Med Hyg 1995;52:117-23.
9. Jenison S, Yamada T, Morris C, Anderson B, Torrez-Martinez N, Keller N, Hjelle B. Characterization of human antibody responses to Four Corners hantavirus infections among patients with hantavirus pulmonary syndrome. J Virol 1994;68:3000-6.
10. Rawlings JA, Torrez-Martinez N, Neill SU, Moore GM, Hicks BN, Pichuantes S, et al. Cocirculation of multiple hantaviruses in Texas, with characterization of the S genome of a previously-undescribed virus of cotton rats (*Sigmodon hispidus*). Am J Trop Med Hyg 1996;55:672-9.
11. Hjelle B, Jenison S, Torrez-Martinez N, Herring B, Quan S, Polito A, et al. Rapid and specific detection of Sin Nombre virus antibodies in patients with hantavirus pulmonary syndrome by a strip immunoblot assay suitable for field diagnosis. J Clin Microbiol 1997;35:600-8.
12. Swofford DL. PAUP: phylogenetic analysis using parsimony [computer program]. Version 3.1.1. Champaign (IL): Illinois Natural History Survey;1991.
13. Nolte KB, Feddersen RM, Foucar K, Zaki SR, Koster FT, Madar D, et al. Hantavirus pulmonary syndrome in the United States: a pathological description of a disease caused by a new agent. Hum Pathol 1995;26:110-20.
14. Hjelle B, Torrez-Martinez N, Koster FT, Jay M, Ascher MS, Brown T, et al. Epidemiologic linkage of rodent and human hantavirus genomic sequences in case investigations of hantavirus pulmonary syndrome. J Infect Dis 1996;173:781-6.
15. Bharadwaj M, Botten J, Torrez-Martinez N, Hjelle B. Rio Mamoré virus: genetic characteristics of a newly-recognized hantavirus of the pygmy rice rat *Oligoryzomys microtis* from Bolivia. Am J Trop Med Hyg. In press 1997.
16. Duchin JS, Koster FT, Peters CJ, Simpson GL, Tempest B, Zaki SR, et al. Hantavirus pulmonary syndrome: a clinical description of 17 patients with a newly recognized disease. N Engl J Med 1994;330:949-55.
17. Khan AS, Gaviria JM, Rollin PE, Hlady WG, Ksiazek TG, Armstrong LR, et al. Hantavirus pulmonary syndrome in Florida: association with the newly identified Black Creek Canal virus. Am J Med 1996;100:46-8.
